# American Dental Association

**Published:** 1892-11

**Authors:** 


### Reports of Society Meetings.

### AMERICAN DENTAL ASSOCIATION.

### *First Day.—Evening Session.*

(Continued from page 764.)

*Dr. Brophy.*—The next matter on our programme will be the presentation of a case by Dr. Cryer, of Philadelphia, on a new operation for the excision of the inferior dental nerve.

*Dr. Cryer.*—I wish to report a case of the dissection of the inferior dental nerve, which I think is new. We have many operations for this nerve, and, as I have no models here, I cut out this piece of pasteboard to represent the jaw. [Illustrating.] On the inside of the jaw we have the inferior dental foramen; on the outside the mental foramen, and from that the canal known as the inferior dental canal, in which pass the inferior dental nerve and the artery; also the canal known as the mylo-hyoid canal, which accommodates the artery and the nerve of the same name. In neuralgia of the mouth and lower jaw, this nerve has been torn out in various ways, an operation, I have no doubt, the members of the Association are acquainted with. By opening the cheek and turning it up, the canal can be reached. By drilling or taking a piece of bone from this the nerve has been drawn from the anterior and from the posterior portion, taking out as much as possible. Another operation is dividing the masseter muscle and trephining, making a round opening, and taking out the nerve; but that has failed very often on account of the nerve getting into the floor of the mouth, which requires that the nerve shall be cut at the bifurcation. Operations by the late Dr. Joseph Pancoast have been to remove the coronoid process entirely; of course that will destroy the use of the temporal muscle, because it will never unite again. It was also found that the cushion of fat which lies under the cheek is often in the way. It was suggested, two years ago, in the Hospital of Oral Surgery, instead of cutting away the coronoid process, to take out the base of the sigmoid notch,—in other words, to deepen it. An opening is made on the face, over the masseter muscle, avoiding the duct of Steno. If that is cut, there may be trouble. Turn the flap upward, and, having it held by an assistant, cut into the ramus of the jaw, using a Kirk chisel, or any instrument with which you can scrape the periosteum off the bone. It can be done with a saw, but that is crude. Take a spear-pointed drill, not a very sharp one, and perforate this full of holes, just as I take these scissors, and then by using a large fissure-burr, intended for surgical work, cut this portion out, which I will illustrate by doing it with the pair of scissors. Operating under antiseptics, it is well to have a bottle suspended four or five feet above, and by adding a tube the flow can be controlled by pressing on the nozzle. Let this run on the burr as it works. That will clear the chips away, and a clear view will be obtained just beyond the instrument. When the nerve, artery, and vein are struck, it will be known. The burr will rub against these without injuring them, and when the centre of the bone is reached you are opposite the foramen. If satisfied thus far with the operation, the nerve is separated from the vessels. It is best not to destroy these, on account of possible hemorrhage. By using forceps with a gauge, the nerve can be seized and the instrument will retain its hold. The mylo-hyoid nerve runs in this direction. Take as much of it out as possible. It is said that it can be pulled out. I do not think it can, because the force is drawing against every fibre that goes up into the different teeth, and the result will be that it will be broken. If broken, it has been pulled out and still held in the forceps. Perhaps an inch of the nerve is out, including the mylo-hyoid nerve. As the assistant holds this, take an instrument with a hook on it, and push it up towards the base of the brain, under the external pterygoid muscle. Push it along as far as possible, and then cut it off.

*Dr. Patrick.*—I would like to ask the gentleman who the surgeon was that advocated the process described to reach the inferior dental nerve.

*Dr. Cryer.*—I think it was Dr. Pancoast.

*Dr. Patrick.*—How could you amputate a portion of the ramus and the neck of the condyle under the process without having the zygomatic arch interfere?

*Dr. Cryer.*—The zygomatic arch did not interfere. It was cut in a direction like this [illustrating], either by a circular saw or the ordinary Hey's saw. A gentleman asked me if this cured neuralgia. The operation has been performed twice,—the first a year ago last March, and the last in March, a year apart. Both patients are entirely well. The first one has a little twinge occasionally in the region of the tip of the tongue, which may come from another nerve, but he is much relieved and very comfortable.

On motion of Dr. Brophy, the courtesy of the floor was extended to practitioners in medicine.

*Dr. A. Hoadley.*—I have listened to the description of this so-called new operation for the excision of the inferior dental nerve with much interest. There are some new points about it, inasmuch as it contemplates removing the sigmoid notch. Other than that, I do not think there is anything unusual in the operation. It very often happens that when the old operation of trephining over the inferior dental canal is undertaken, the bridge of bone that exists above that is frequently snipped out for the purpose of giving more room and then cutting off the nerve about the mylo-hyoid. Pulling out the inferior dental nerve is sufficient to destroy the function of the nerve. If it is drawn out from the inferior dental canal, there will certainly be no further neuralgia from that point. Its peripheral function is destroyed. Should neuralgia continue to exist over the branches of the nerve, it is positive that the irritation exists higher up, possibly where the nerve passes out of the skull at the foramen ovale.

There is one other point I wish to speak of in regard to the anatomy of the parts. The coronoid process only rises slightly above the sigmoid arch, to which the temporal nerve is attached. It can be cut off far below the arch, and it can be united, after having been divided, with excellent prospects of union. If I remember rightly, the gentleman who described the operation stated that it was objectionable to divide the coronoid process, but, if I am not mistaken, if good apposition is made and the fragments secured by suture, a good union is accomplished. In fractures of the under jaw union is made without difficulty. It is a bone that unites, as a rule, under almost any circumstances, but once in a while a diseased process will give some trouble by perhaps interfering more with nutrition than with the factor of non-union. The operation is one that is extremely simple. It is not attended with any danger, and it can be performed exactly according to the methods laid down by the gentleman who described them; the nerve can be cut off a little higher up by this method than by the old one of trephining; but the difference at the point of the upper part of the mylo-hyoid and the upper part of the sigmoid arch does not make any change in neuralgia. The difference between the division of the mylo-hyoid and the highest point described by the gentleman would be of little consequence in the curative effect of the operation. I thank you for the privilege of speaking.

*Dr. Morris.*—I am very much surprised to see this subject brought up in the form of an unpleasant operation from the outside, when we have the method which was first introduced by Professor Hutchins, of using the dental drill on the superior surface of the dental arch, making two or often only one cut through the upper part of the jaw-bone, down upon this canal, cutting it off at any desired point, and not making a wound upon the external surface larger than a quarter of an inch, and giving as much relief as any of these surgical operations have ever afforded. I cannot understand why this method is not more extensively used by the dental profession. It was given to the public by Professor Hutchins about 1875. I furnished the dental drills that were used with the method. Professor Hutchins was the first one to use it, and now the old method is preferred, making unsightly scars.

*Dr. Fillebrown.*—I do not make any wound on the external surface. It seems to me that, as there is not one case in a hundred that proves a permanent cure, it is hardly worth while to go to such an extensive disfiguration of a patient as opening from the outside of the face. It is possible to remove it within a quarter of an inch of the point of the operation described, and all within the mouth. I have a patient who to-day has the inferior dental nerve removed from the second inferior bicuspid, which is still in the mouth, to almost three-quarters of an inch beyond the foramen, where the nerve enters the inside of the jaw. She suffered with neuralgia. I trephined and removed three-quarters of an inch back of the second bicuspid. She was well for a year and a half. Soon after that she came, and I performed another operation. I cut back through the canal, and took it all out right into the soft tissue. She afterwards insisted that I should go farther, and, although there was not much hope, I did it to please her. The second operation cured her for six months. The last operation did very little good, and to-day she suffers very much. The trouble is central where the nerve emerges, or it may be still more central than that. The point I wanted to make is, that to remove the nerve almost to the foramen it is entirely unnecessary to disfigure the exterior of the face, but it may be done readily through the mouth.

*Dr. Brophy.*—I have never had occasion so far to make an external incision for the removal of the inferior dental nerve. The operation of removing the dental nerve within the mouth I think can be accomplished in every case where the operator may be successful in approaching the posterior surface of the second molar for the insertion of a filling. The operation which Dr. Cryer describes is a new one, but it seems to me that the external incisions may be avoided, and should be avoided where it is possible to do so. If we had to go to the centre, to the base of the brain, for the purpose of dividing the nerve, it would be necessary to make an external incision ; but to proceed in such a way as to prevent the neuralgia from recurring, is to remove the inner portion of the canal, so as to induce an exudation which will fill it, and this we may do with a strong drill, carrying it backward along the canal and arch, seize the nerve with the forceps, and draw it down to a point where we may be able to hold it. If we do this, and then ream out the canal, and keep it cleansed antiseptically, in a short time it will be filled with new tissue, and the neuralgia consequently will not recur. The old operation of seizing the nerve by carrying it backward and moving it forward may be done, and the nerve divided in that manner; but the danger of hemorrhage should cause a hesitation to perform this latter. I think it is always well to first make the operation within the mouth, and then, if we find it necessary to extend it further, the external incision should be made as a last resort.

*Dr. Cryer.*—I hope those present will not think that the gentleman with whom I am associated in surgical procedures would undertake this operation for neuralgia of the lower jaw without first testing some of the minor operations: he would not think of cutting into the cheek until the others had been tried. We all know that after the removal of even two inches of nerve there will be a union of that nerve. In my own body I have had two and a half inches of nerve removed and the rest united, and I have seen canals by more than one surgeon cleaned from the mental foramen as far back as it is possible to go and the nerve removed, and in a very short time the neuralgia returned. One of the gentlemen spoke of taking a tenaculum and passing it in at the inferior dental foramen on the inside, catching it there, and drawing the nerve out and cutting it. That is a very nice operation; but the mouth has to be propped open, the patient must receive ether through the mouth, the sponges and the napkin are in the way, and you have arteries in that region that are very dangerous when cut. I assisted at one of those operations, and we had a serious time to save the patient's life. I do not know which artery was cut. The patient almost choked with blood rushing into the mouth. It was finally checked, but has remained as a warning against undertaking that operation again. You cannot get back and perform it in the mouth; the patient is liable to suffocate, and may bleed to death without showing any external sign.

As late as five years ago, operations on the face made a bad scar. To-day these can be made, and in two weeks' time you cannot see a mark. Of course, as I have said before, I would not undertake to do this until the minor ones had failed.

The idea of reporting this case was that it was only new as extending the sigmoid arch, and it opens it immediately above the nerve, and the nerve comes directly in view and can be easily taken hold of. I want to claim the advantage of cutting the nerve off there. Many claim that it is purely a motor nerve coming from the motor branch of the inferior maxilla, but my experience in the last twelve years in hospitals shows me that it is best to destroy the nerve and to remove as much of it as possible. There may be new bone, and if you can get to the ganglion and cut it off, so much the better.

Dr. Alton H. Thompson, of Topeka, Kan., then read a paper, entitled "The Grinding Teeth of the Herbivorous Mammalia," an abstract of which follows:

The molars of the herbivorous mammalia present an interesting and remarkable example of a high degree of specialization. The complex structure of the masticating surface of those teeth is produced by an arrangement of the dental tissues in alternating layers, the different densities of the enamel, dentine, and cementum allowing of different degrees of wearing away, so that an irregular surface is constantly presented for the purpose of triturating resisting vegetable fibre, which is such an important function in the physiological economy of the vegetable-feeders. In use, the enamel, being hardest, stands highest; the dentine, being less dense, stands lower; and the cementum, being softest, stands lowest of all. The molars in the ruminants and all vegetable-feeders are formed as though layers of enamel, dentine, and cementum had been laid upon each other and the whole then rolled and crushed together. This produces a very effective instrument for mastication, and is a highly specialized form of tooth, as we shall notice later. A cross-section of a tooth reveals a pattern which presents a great variety in different species and is of distinct diagnostic value. This pattern differs in different families and in different orders, but is uniform in a species, and is closely related in each genus. By the study of this pattern, naturalists have a means of diagnosticating species, and it is of great value to the palæontologist in the classification of the remains of extinct animals.

The teeth are the most durable of the animal tissues, and form the most constant medium for the identification of fossil remains. In the evolutionary history of the horse, which was so beautifully worked out by our brilliant palæontologists, the testimony of the teeth was necessary for the confirmation of the descent of the species. It is satisfactorily proven that the single-toed horse of to-day is descended from five-, three-, and two-toed ancestors, who had all the characteristics of the horse, even to the molar pattern. So the biological history of other groups has been worked out with more or less certainty, and the teeth have performed an important part in the evidence they have furnished. In the vegetable-feeders the characteristic patterns of the molar teeth have been reliable indications of affinities, on account of their uniformity. As Owen again says: "Different groups of the ungulata, not only orders and genera, but even species, are characterized by the various patterns which result from the various forms, directions, and proportions in which enamel and cement alternate with dentine in the substance of the crowns of the complex molars."

The most highly specialized group is that of the ruminants, of which Owen says: "In the ruminants the characteristic complexity of the molar teeth is manifested even in the deciduous series, but in the permanent series only by the three posterior teeth of both upper and lower jaws, which are the true molars. The complexity is the result of peculiar folds of deformative capsules, some of which are longitudinal, or project inward from the side of the capsule and form peninsular folds of enamel upon the grinding surface of the teeth, while others depend vertically from the matrix into the body of the tooth, and form islands of enamel when the crown begins to be worn. Of the longitudinal folds, two in the upper true molars are external, broad but shallow, and often sinuous, and one is internal, narrow and deep, and extending quite down the summit of the crown, and decreasing in depth towards the end of the crown. The corresponding folds of enamel in the completed teeth accordingly extend more or less across the teeth from within outward, as the tooth is more or less worn. The crown of the tooth is thus divided into two parts, one in front of the other, the inner side of each lobe being convex, the other concave in a less degree, and usually with a convex prominence in the middle.

"The vertical folds of the capsule, two in number, extend one into each lobe, and in the upper molars are concave towards the outer and convex towards the inner side of the lobe, which is thus divided into semicylindrical lobules with crescentic summits, as seen on a transverse section or on the grinding surface of the tooth. Each lobule consists of a middle bay of dentine, and an outer coat of enamel and thin cement. The midspace between the lobules—the centre of the vertical folds—is filled with cement, and commonly contains portions of vegetable fibre. It is inclosed by a crescentic island of cement, and the whole of the circumference of this complex molar is also invested by a coat of enamel, and this by a layer of cement. Different genera of the ruminants also differ in the depth and sinuosity of the two outer longitudinal folds, and in the depth and complexity of the two vertical folds, which likewise are united in some species by a longer column than in the others."

The origin and evolution of the teeth of the plant-eaters form a most interesting study. Being highly specialized, they have arrived at a high degree of perfection by gradual steps, most of which can be traced in the successive steps furnished by both living and extinct forms. As Professor E. D. Cope says ("Origin of the Types of the Molar Teeth," etc.): "The simple tubercle may be regarded as the least specialized form of tooth, and the transition from simple to complex teeth is accomplished by the repetition of the type of the simple tooth in different directions.

"Complex teeth, as the tubercular molars, merely exhibit additional lateral tubercles, and sometimes additional longitudinal ones. The former is done in two ways,—*i.e.*, either by the folding of the sides or by the development of tubercles on the crown. Upon this basis are constructed the more complex types of teeth exhibited by the various families of the ungulata and some rodentia. The crowns of the superior molar teeth of the higher mammalia are supported on three roots, two of which are external and the third internal. The corresponding inferior molars are supported on two roots, and are less complex, but those two roots usually support four tubercles, two on each side, while the roots of the superior molars support directly but one each. The crown of the inferior molar is, therefore, more complex than that of the other. It divides the different kinds of molars into special classes, as follows:

"1. The haplodont, in which the crown is undivided and simple, low and obtuse as in the cetacea, or acute as in the carnivora.

"2. The ptychodont type, the crown folded upon the sides, the folds frequently crossing the crown, as in the rodentia.

"3. The bunodont type, the crown supporting tubercles, which are few and opposite or alternating, and may be many and regular. To this class belong the mastodon, man, the bear, the carnivora, and some rodentia.

"4. The lophodont type, the summit of the crown thrown into folds of transverse or longitudinal direction, as in the higher ungulates. It embraces the many types of ungulates, some rodentia, and others.

"The transition between the bunodont or tubercular teeth and the lophodont or folded teeth is very obvious, so as to lead to the belief that the several subdivisions of the lophodonts represent modifications of corresponding types of bunodonts. Intermediate forms are seen, where the tubercles of the mandibular teeth are compressed, while they remain conic.

"Thus the teeth of the hippopotamus, whose molars present four principal tubercles, are truly bunodont; the valleys separating the tubercles are deeper than the truly bunodont genera, but much shallower than those of the true lophodonts. In the latter, the valleys are deepened and the tubercles are more compressed and elevated, as in the deer. In the ancient species of deer the crests are lower and the valleys shallower than in the living species. The lophodont molar was produced by modification of the bunodont, and the manner of the change has been by constant acceleration of the growth of the folds of the crown upward, and perhaps downward in its long axis, and acceleration of the length of the crown."

Or he again says ("The Extinct Mammalia"): "The ordinary tooth of the higher type of the mammalia, whether hoofed or not, with some exceptions, is coupled with crests or cusps. By cutting the complex grinding surfaces we find that they have been derived by the infolding of extensions of four original cusps or tubercles. They have been flattened, have been rendered oblique, have run together, have folded up, have become acute, have descended deeply, or have lifted themselves, so that we have teeth of various kinds, sometimes very elegant, and ofttimes very effective in mechanism. In many primary ungulates the primitive condition of four conical tubercles is found, and in passing to older periods we find the mammalia of the Puerco period, which rarely have more than three principal tubercles. In the succeeding periods, however, they get the fourth tubercle and then others, and finally a complicated grinding or cutting apparatus."

Extensive study has convinced the naturalists and evolutionists of our day that the Lamarckian theory of use and disuse, or, as it is latterly called, the mechanical effects of use, accounts for the principal influence in the development of tooth forms.

Dr. John Ryder, in his article on "The Mechanical Genesis of Tooth Forms" ("Proceedings of the Academy of Natural Sciences," Philadelphia, 1878), has elaborated and described this fully, and shown how the theory accounts most beautifully for the origin and formation of many forms of teeth, and especially of the class we are now studying. He says: "Amongst the ungulata the most conclusive evidence is found of dental modification. The numerous living and extinct forms present a remarkable chain of dental forms, gradually developing from the bunodont type and passing into the exclusively modified selenodont type, acquired by increased mobility of the mandible.

"In elephas and mastodon the jaw movement is from behind forwards: the molars are tuberculate in crown crests, which become obsolete from wear and expose the transverse plates."

The antero-posterior movement has developed the form which offers the greatest resistance to this movement in mastication and consequent efficiency in reducing food. The primitive form of jaw-movement is that of simple opening and closing, as in the typical carnivora. Next came the backward and forward movement, as in the proboscidæ and other primitive types, and then the lateral movements of the jaw, and finally the extensive triangular excursions of the jaw of the ruminants and other vegetable-feeders, as the highest of all.

"The opening and closing movement is more characteristic of dogs, cats, pigs, hippopotami, bats, opossums, and all other homodonts or their immediate allies. The pigs show the first tendency to lateral movements, and have lengthened crests to oppose this movement of masticating. The tapir presents still more lateral movement, and the kangaroo still more. The rhinoceros describes a much larger excursion, and the ruminants the largest of all. In these the lateral is quite extensive, if not more so than the longitudinal movement. In all these forms there is, of course, corresponding modification of the condyle and glenoid cavity to permit of the typical movement. Indeed, the development of the peculiar movement of each class has modified the articulation as it has the teeth. The forms of teeth result from the strains incident to mastication. Thus, in lepus the strain is upon the inner side of the upper molar, and on the outer surface of the lower molar. This leads to a peculiar straining and curving of the teeth, and a corresponding arrangement of the dental tissues, the recurve pattern of the opposing molar series simulating most closely some of the mechanical devices used by men in grinding-mills."

In cervus the idea of "displacement due to strain" is shown most remarkably. There is some evidence that the premolars are acted upon from the external side as well as from the internal, which tends to keep the jaw narrow at that point. The true molars are, however, acted upon from the inside on the upper jaw, and from the outside on the lower jaw. This tends to spread the jaw at those points, and modify the enamel pattern. The motion being lateral, the strain both on tooth and tissues must be very powerful. In the giraffe, the molar line of the lower series is slightly convex internally, and it has extensive lateral excursion of the mandible. The upper series is convex internally. The displacement seems to be greatest where the masticating muscles act with greatest force. In the rodents there is a reciprocating motion, which counteracts the lateral, so that displacement is not very great. In cervus there are deep transverse valleys and cross-crests, showing the persistence of this arrangement where the mandibular movement is entirely lateral.

"It looks as though the strain incident to mastication had pressed the cusps of the tooth flat, and curved their cornus outwards in the upper series and inwards in the lower by their oft-repeated excursions in one direction. The flexures are modified in accordance with the various directions of the excursion forces, and the plicate layers of the dentine of the horse, and perhaps of the ox and deer, may be accounted for in the same way. The plication is greatest in a line parallel with the direction of the strain exerted during mastication. Another circumstance is the greater inclination of the inner cusps of the upper molars outwards, and of the outer ones below inwards, which is so common among selenodont ungulates. The mandibular apparatus of mammals may be regarded as a lever of the third class, in which the glenoid cavity is the fulcrum, the maxillary force the power, and the resistance of food to the teeth in crushing the weight. Since the average of muscular force is one hundred and four pounds to the square inch of transverse section, it is easy to surmise what would be the tendency of the force of mastication. It is not all expended in reducing food, but must react upon the structures which are subjected to the strain."

But we cannot follow this interesting study further; enough has been given to show the origin and the method of evolution of the remarkable and complicated structures of the molars of the herbivorous mammals. The great influence dictating this unique formation is food-selection, which is so powerful in modifying the masticating apparatus of animals. In no field is the study of tooth formation and its evolution more interesting than in that of this class, on account of the highly specialized forms that have been developed in obedience to a demand for a more effective apparatus for the reduction of refractory foods.

(To be continued.)

